# The relationship between H2FPEF score and contrast induced nephropathy in patients with ST elevation myocardial infarction

**DOI:** 10.34172/jcvtr.2022.30537

**Published:** 2022-12-24

**Authors:** Ufuk Sadik Ceylan, Ersin Yıldırım

**Affiliations:** ^1^Istanbul Dr. Siyami Ersek Education and Research Hospital, University of Health Sciences, Department of Cardiology, Istanbul, Turkey; ^2^Istanbul Umraniye Education and Research Hospital, University of Health Sciences, Department of Cardiology, Istanbul, Turkey

**Keywords:** Contrast Induced Nephropathy, Myocardial Infarction, Percutaneous Coronary Intervention, H2FPEF Score

## Abstract

***Introduction:*** In the present study, we aimed to investigate the relationship between H2FPEF score and Contrast Induced Nephropathy (CIN) in patients with myocardial infarction with ST segment elevation (STEMI).

***Methods:*** A total of 355 patients who had been diagnosed with ST elevation-myocardial infarction and undergone primary coronary angioplasty were retrospectively included in the study. The patients were divided into two groups according to the presence of CIN and these groups were compared in terms of baseline characteristics and laboratory findings. The H2FPEF score was calculated for each patient on admission and later compared between the groups.

***Results:*** The distribution of the study population was as following: 63 (17.7%) CIN (+) and 292 (82.2%) CIN (-). In CIN (+) group, the mean H2FPEF Score (2.00±1.60 vs 1.25±1.26, *P*<0.001) was significantly higher than the CIN (-) group. H2FPEF Score (OR: 1.25, 95%CI: 1.01-1.55), and mean age (OR: 1.03, 95%CI: 1.00-1.06) were found to be independently associated with CIN development.

**Conclusion:** H2FPEF score is an independent predictor of CIN development in patients with acute STEMI. It is easily calculated and and may be used to estimate the CIN in STEMI patients.

## Introduction

 Contrast-induced nephropathy (CIN) is a clinical form of acute renal injury, which typically occurs after the intravascular injection of iodinated radiographic contrast media and despite being one of the major causes of iatrogenic acute renal failure, mechanism of CIN has not yet been clearly understood. The possible mechanisms of CIN are medullary ischemia, oxidative stress, vasoconstriction, and direct toxic effects of contrast agents. CIN is associated with increased mortality, contributes to morbidity and prolonged hospitalization.^1,2^ The incidence of CIN has decreased in recent years thanks to the use of less nephrotoxic contrast agents and better prevention strategies. However, CIN development after coronary angiography still persists as one of the most important causes of mortality and morbidity,^3,4^ especially among the patients treated with primary PCI, rather than who undergo elective PCI.^5^ Therefore, estimating the risk of developing CIN in patients presenting with acute coronary syndrome is still clinically important.

 H2FPEF score, which utilises clinical and echocardiographic characteristics that are obtained in the evaluation of patients with unexplained exertional dyspnea, enables discrimination of heart failure with preserved ejection fraction (HFpEF) from non-cardiac etiologies of dyspnea. The six clinical and echocardiographic determinants that is utilized to calculate the H2FPEF score are; a body mass index (BMI) > 30 kg/m^2^ (H); the use of ≥ 2 antihypertensive drugs (H); the presence of atrial fibrillation (AF) (F); pulmonary hypertension which is defined as a systolic pulmonary arterial pressure (PAP) of > 35 mm Hg (P); an age of > 60 years (E); and elevated filling pressures evident from E/e’ > 9 (F). The presence of paroxysmal or persistent AF yields 3 points, BMI > 30 kg/m^2^ yields 2 points, and all of the other criteria listed above yield 1 point.^6,7^ HFpEF gets more probable as H2FPEF score increases.^8^ However, due to the presence of some common risk factors, it has been researched to be associated not only with HFpEF, but also with other cardiovascular problems such as coronary artery disease and arrhythmia.^9,10^ Thus, we hypothesized that H2FPEF score system may be a powerful tool in the determination of the probability of a kidney function deterioration and progression into CIN in STEMI patients before commencing the needed invasive treatment.

## Materials and Methods

###  Patient population

 After the Ethics Committee approval, a total of 355 patients who had been admitted to our hospital due to acute STEMI and undergone primary coronary intervention were retrospectively included in the study. The patients were divided into two groups according to the presence of CIN and than these groups were compared in terms of baseline characteristics and laboratory findings. The clinical and demographic characteristics of the patients were recorded. The inclusion criteria included (a) STEMI (b) undergoing primary cardiac intervention (angioplasty and/or stent implantation). For STEMI, the following diagnostic criteria were used: 1. ST segment elevation in ≥ 2 consecutive derivations (in chest derivations ≥ 2 mm and in extremity derivations ≥ 1 mm) or new-onset left bundle brunch block (LBBB), 2. Ischemic type chest pain lasting more than 30 minutes, 3. An elevation in serum creatine phosphoki­nase myocardial band (CK-MB) and troponin levels at least 2 fold of more than maximum reference value. The Killip class was evaluated as follows; class 1, no evidence of heart failure (HF), class 2, signs indicating mild to moderate degree of HF, class 3, pulmonary edema, and class 4, cardiogenic shock or hypotension. A 12-derivation ECG record was obtained for all patients just after admission. The blood samples were obtained at the time of admission and during the follow-up (Coulter LH 780, Beckman Coulter Ireland Inc., Mervue, Galway, Ireland). Echocardiography examination was performed by an experienced cardiologist at the coronary intensive care unit just after the primary PCI (Vivid 5 system, Vingmed GE, Horten, Norway) in all study participants and the left ventricular ejection fraction was calculated using the modified Simpson method. Contrast-induced nephropathy was defined as 25% or higher elevation in the basal creatinine value or 0.5 mg/dl or higher elevation in the creatinine value.

###  H2FPEF score

 For each patient, the H2FPEF score was calculated by an experienced cardiologist. Six determinants of H2FPEF score, which are obesity (BMI > 30 kg/m^2^) (2 points), atrial fibrillation (3 points), age > 60 years (1 point), hypertension with a need for 2 or more antihypertensive drugs (1 point), E/e’ > 9 (1 point) and pulmonary arterial systolic pressure > 35 mm Hg (1 point) were evaluated in the study population. H2FPEF score ranged from 0 to 9 points.

###  Coronary Angiography and In-hospital follow-up

 Percutaneous coronary interventions were performed via the femoral route by an experienced cardiologist (Siemens Axiom Artis Zee, Germany). Nonionic low-osmolality contrast medium (Omnipaque 350 MG/ml; GE Healthcare, Cork, Ireland) was used for coronary interventions. Images were recorded in multiple projections for the left and right coronary arteries. All patients were given 300 mg aspirin with an addition of either 600 mg clopidogrel or 180 mg ticagrelor loading dose prior to the procedure. 100 U/kg intravenous heparin was administered to each patient after having visualized the arterial anatomy. Glycoprotein IIb/IIIa use was left at the discretion of the physician. After the patients were transferred to the intensive care unit, treatment continued with 100 mg aspirin with either 75 mg clopidogrel or 90 mg ticagrelor bid. The decision for concurrent use of angiotensin converting enzyme inhibitors, beta-blockers and statins was made according to the recommendations of the American College of Cardiology/American Heart Association. Blood pressure and electrocardiogram monitoring were performed at the intensive care unit and control blood samples were obtained. Use of nephrotoxic agents and non-steroidal antiinflammatory drugs was avoided. Oral fluid intake was re-initiated 90 min after the procedure for the patients with good general status. Patients who did not have congestive heart failure were administered 1 mL/kg/h of IV 0.9% isotonic saline solution for 24 hours. The patients were followed-up with plasma creatinine levels during 72 hours after the procedure.

###  Statistical analysis

 Statistical analysis performed using SPSS 22.0 for Windows Evaluation Version statistical package. The normality distribution was evaluated using the Kolmogorov-Smirnov test. Continuous variables were presented as mean ± standard deviation. Categorical variables were summarized as frequencies. Differences between the two groups according to continuous variables were determined by the independent samples t-test. Categorical variables were compared by, chi-square or Fisher’s exact test. The logistic regression analysis was used for determining the effect of potential prognostic factors on the presence of CIN, and the independent predictors were determined through inclusion of significant risk factors in the logistic regression model. A p level of < 0.05 was accepted as statistically significant with 95% confidence interval and 5% margin of error.

## Results

 The distribution of the study population (n = 355, mean age 56.87 ± 10.94) was as following: 63 (17.7%) CIN ( + ) and 292 (82.2%) CIN (-). The proportion of women in the whole study group was 16.9%. There was no significant difference was observed in terms of gender ratio. The mean age was significantly higher in the CIN ( + ) group compared to the CIN (-) group (61.31 ± 11.04 vs 55.91 ± 10.70; *P* = 0,001). The rates of hypertension (49.2% vs 35.3%, *P* = 0.039) and atrial fibrillation (11.1% vs 4.1%; *P* = 0.025) were higher in the CIN ( + ) group compared to the CIN (-) group. Mean body mass index (28.26 ± 3.55vs 27.18 ± 3.78; *P* < 0,039) was detected significantly higher in the CIN ( + ) group. No differences were observed between the groups in terms of other demographic characteristics. The blood urea nitrogen level (36.12 ± 9.15 vs 33.37 ± 10.05; *P* < 0,047) was significantly higher in the CIN ( + ) groups compared to the CIN (-) group. No difference was observed between the groups with regard to other laboratory findings (Table 1). In CIN ( + ) group, the mean H2FPEF Score (2.00 ± 1.60 vs 1.25 ± 1.26, *P* < 0.001) and KILLIP score (1.38 ± 0.95vs 1.11 ± 0.53; *P* < 0.040) were significantly higher than the CIN (-) group.

**Table 1:** Baseline Characteristics of the Patients With and Without Contrast-Induced Nephropathy

**Variables**	**CIN (-) (n:292)**	**CIN (+) (n:63)**	***P***
**Baseline characteristics**			
Age (years), mean (± SD)	55.91 ± 10.70	61.31 ± 11.04	0.001*
Gender (female), n (%)	54 (18.7%)	6 (9.5%)	0.080
Current Smoker, n (%)	159 (55.8%)	34 (55.7%)	0.817
Diabetes Mellitus, n (%)	82 (28.8%)	17 (27.9%)	0.887
Cerebrovascular Disease, n (%)	12 (4.2%)	2 (3.3%)	0.737
Left ventricular ejection fraction, (%; ± SD)	47.26 ± 11.07	47.09 ± 10.36	0.925
Previous PCI, n (%)	19 (6.5%)	6 (9.8%)	0.357
Previous CABG, n (%)	7 (2.4%)	2 (3.3%)	0.691
Heart valve disorder	3 (1%)	2 (3.4%)	0.204
Body Mass Index (kg/m^2^; ± SD)	27.18 ± 3.78	28.26 ± 3.55	0.039*
Systolic Blood Pressure (mm Hg; ± SD)	125.826 ± 23.64	127.74 ± 25.22	0.589
Diastolic Blood Pressure (mm Hg; ± SD)	76.95 ± 15.61	78.69 ± 16.03	0.431
Contrast Amount (cc ± SD)	192.13 ± 62.17	205.12 ± 84.51	0.162
**Laboratory Findings**			
Glucose (mg/dL; ± SD)	164.08 ± 77.39	171.15 ± 77.52	0.511
Sodium (mmol/dL; ± SD)	139.65 ± 6.18	138.98 ± 4.45	0.317
Potassium (mmol/dL; ± SD)	4.20 ± 0.44	4.13 ± 0.57	0.689
Calcium (mg/dL; ± SD)	9.19 ± 0.51	9.41 ± 0.48	0.990
Magnesium (mg/dL; ± SD)	2.15 ± 0.20	2.20 ± 0.23	0.132
BUN (mg/dL; ± SD)	33.37 ± 10.05	36.12 ± 9.15	0.047*
Creatinine (mg/dL ± SD)	0.90 ± 0.22	0.82 ± 0.33	0.103
HDL-C (mg/dL; ± SD)	38.84 ± 9.22	37.96 ± 9.43	0.523
LDL-C (mg/dL; ± SD)	123.19 ± 39.70	119.15 ± 34.23	0.454
Triglyceride (mg/dL; ± SD)	163.22 ± 84.99	161.07 ± 73.16	0.859
WBC (x10^3^ /µL; ± SD)	12.65 ± 12.54	11.98 ± 3.51	0.673
Hemoglobin (g/dL; ± SD)	13.90 ± 1.50	13.96 ± 2.20	0.831
Hematocrit, n (%; ± SD)	39.80 ± 4.28	40.20 ± 5.40	0.577
Platelets (x10^3^ /µL; ± SD)	251.16 ± 66.77	263.71 ± 65.93	0.176
Troponin (ng/mL; ± SD)	22.31 ± 33.66	23.77 ± 31.92	0.840
Peak CK-MB (U/l; ± SD)	179.74 ± 126.17	187.31 ± 181.83	0.700
**Risk Scores**			
H2FPEF Score ( ± SD)	1.25 ± 1.26	2.00 ± 1.60	0.001*
Body Mass Index > 30kg/m^2^	68 (23.3)	19 (30.2%)	0.250
Hypertension, n (%)	103 (35.3%)	31 (49.2%)	0.039*
Atrial Fibrillation, n (%)	12 (4.1%)	7 (11.1%)	0.025*
Pulmonary Hypertension	27 (9.2%)	12 (19%)	0.024*
Elder (Age > 60 years)	112 (38.4%)	31 (49.2%)	0.111
Filling Pressure (E/e’ > 9)	38 (13.0%)	10 (15.9%)	0.547
KILLIP Score ( ± SD)	1.11 ± 0.53	1.38 ± 0.95	0.040*

Abbreviations: BUN, blood urea nitrogen; HDL-C, high density lipoprotein cholesterol; LDL-C, low density lipoprotein cholesterol; WBC, white blood cell; CABG, coronary artery bypass grafting; PCI, precancerous coronary intervention. *Independent Samples T-Test, chi-square Test, Fisher’s Exact Test *P < 0.05statistically significant. Continues variables are reported mean ± SD). Categorical variables are reported n (%).

 Among the demographic and laboratory findings (Table 1) those that were found to be associated with CIN were evaluated as potential risk factors and they were evaluated with the logistic regression analysis. H2FPEF Score (OR: 1.25, *P* = 0.035), and mean age (OR: 1.03, *P* = 0.029) were found to be independently related to CIN development. Level of blood urea nitrogen in hospital admission, hypertension and KILLIP score were not found to be independent predictors of CIN development (Table 2). For CIN complication, all variables of the H2FPEF score were investigated one by one regression analysis. H2FPEF score, which is formed by combination of all variables was found to be an indipendent predictor for CIN (OR: 1.33, *P* = 0.028) (Table 3). In ROC curve analysis, at a cut-off level of 1.5, H2FPEF score predicted CIN with a sensitivity of 64.0% and a specificity of 72.1% (Area Under Curve (AUC): 0.64; 95% CI: 0.56-0.71; *P* < 0.001).

**Table 2:** Regression analysis of potential prognostic factors for the Contrast-Induced Nephropathy

	**Univariable analysis**		**Multivariable analysis**	
**Variables**	**OR (95% CI)**	***P*** ** value**	**OR (95% CI)**	***P*** ** value**
H2FPEF Score	1.43 (1.18-1.72)	< 0.001*	1.25 (1.01-1.55)	0.035*
KILLIP Score	1.60 (1.14-2.25)	0.006*	1.41 (0.97-2.04)	0.069
BUN	1.02 (1.00-1.05)	0.051	-	-
Hypertension	1.77 (1.02-3.07)	0.040*	1.29 (0.71-2.33)	0.394
Age	1.04 (1.02-1.07)	< 0.001*	1.03 (1.00-1.06)	0.029*
Diabetes Mellitus	1.04 (0.56-1.93)	0.887	-	**-**
Gender (female)	2.18 (0.89-5.32)	0.086	-	**-**

Abbreviations: OR, odds ratio; CI, confidence interval. *P < 0.05statistically significant.

**Table 3:** Regression analysis of H2FPEF Score variables for associated Contrast-Induced Nephropathy

	**Univariable analysis**		**Multivariable analysis**	
**Variables**	**OR (95% CI)**	***P*** ** value**	**OR (95% CI)**	***P*** ** value**
H2FPEF Score	1.43 (1.18-1.72)	< 0.001*	1.33 (1.03-1.73)	0.028*
Body Mass Index > 30 kg/m^2^	1.42 (0.77-2.59)	0.252	-	-
Hypertension	1.77 (1.02-3.07)	0.040*	1.50 (0.84-2.68)	0.169
Atrial Fibrillation	2.91 (1.10-7.73)	0.031*	1.13 (0.30-4.26)	0.853
Pulmonary Hypertension	2.30 (1.09-4.85)	0.027*	1.81 (0.81-4.07)	0.810
Elder (Age > 60 years)	1.55 (0.90-2.69)	0.113	-	**-**
Filling Pressure (E/e’ > 9)	1.26 (0.59-2.68)	0.548	-	**-**

Abbreviations: OR, odds ratio; CI, confidence interval. *P < 0.05statistically significant.

## Discussion

 CIN is an important cause of iatrogenic acute renal failure^11^ and anticipating the occurrence of CIN development may give us the opportunity to take necessary measures to prevent renal failure. CIN has been extensively studied since the 1950s due, in part, to its devastating adverse events but the mechanism of CIN has not been able to be understood completely yet. Reactive oxygen species induced combined hypoxic and toxic injury is important for CIN development.^4,12^ There are also studies indicating that contrast agents reduce the renal blood flow and lead to vasoconstriction in renal arteries.^13^ CIN incidence which varies depending on the population, has a complex underlying pathophysiology and further studies are needed to lift the veil on this matter. In our study, we determined that the H2FPEF score is a independent predictor for CIN in patients with STEMI. To our knowledge, this study is the first to determine the relationship between H2FPEF score and CIN.

 Kabeer MA, et al showed that obesity is a risk factor for contrast induced nephropathy.^14^ The close relationship between atrial fibrillation and CIN has been demonstrated in recent studies.^15,16^ Also it has been demonstrated that hypertension, heart failure, age, nephrotoxic drugs, decreased intravascular volume, play important roles in the development of CIN.^17,18^ As seen here, many important risk factors for CIN are also common parameters within H2FPEF score. This condition suggests that the H2FPEF score may be useful for prediction of CIN. Before this study, Cicek G. et al showed that risk scores developed for different purposes can be useful in CIN estimation.^19^ With this study, we demonstrated the close relationship between the H2FPEF score, a new and updated scoring system, and the development of CIN. CIN rate was determined as 17.7% in our study. Previous studies have shown that this rate is up to 25% in STEMI patients.^20^ The higher incidence in this group may be associated with the high-risk profile of these patients. In addition, timing is important in determining of the CIN because creatinine elevation is relatively slow, requiring 48–72hr to identify many cases of CIN.^21^ CIN incidence may be detected to be low in patients with undergoing elective PCI, due to early discharge of these patients.^22^ Our study has shown that CIN is still common among hospitalized patients. We hope that the development of CIN can be predicted by using the risk assessment scores and the incidence can be reduced with the measures to be taken.

 The H2FPEF score, which relies upon simple clinical characteristics and echocardiographic parameters, enables discrimination of HFpEF from noncardiac causes of dyspnea and can assist in the determination of the need for further diagnostic testing in the evaluation of patients with unexplained exertional dyspnea. The probability of HFpEF gets higher with an increasing H2FPEF score.^8^ Each component of the H2FPEF score is simple, and not only the calculation is quite easy in clinical practice with a low cost, but it is also well validated, which indicates that the score can be widely applied. Previous studies showed that the H2FPEF score, which was originally developed for HFpEF, can be used as a useful predictor in cardiovascular problems other than HFpEF due to common risk factors.^9,23^ However, contrary to these studies, Ravi B Patel et al could not detect any relationship between H2FPEF score and atrial fibrillation recurrence in patients who underwent cryoballoon ablation.^10^ With all these data, new studies are needed to fully reveal the importance of the H2FPEF score in clinical practice.

 In this study, for CIN complication, all variables of the H2FPEF score were investigated one by one via regression analysis. H2FPEF score, which is formed by combination of all variables was found to be a stronger predictor then all its components. As stated above, the relationship between risk factors such as atrial fibrillation, hypertension, age and CIN has been demonstrated in previous studies. Although there are studies on the relationship between E/e’ and CIN, there is no study on the relationship between pulmonary hypertension and CIN to our knowledge.^24^ In addition, the results about the BMI index are inconsistent. While low BMI is believed to be associated with CIN, recent studies have shown that obesity is a risk factor for CIN.^14^ In this study, we revealed that the H2FPEF score obtained by combining all these variables can help us more in predicting the risk of CIN. On the other hand, surprisingly, diabetes was not identified as a risk factor in this study. This would contradict some of the previous studies in the literature, but the limited number of patients in the study and the restriction of metformin use may have contributed to this.

 Our study has revealed that high H2FPEF score levels on admission can predict CIN. The H2FPEF score may be helpful as it may be calculated rapidly and easily. The H2FPEF score can help the clinician to predict the development of CIN without waiting for any blood test results. This may provide great advantage for clinicians to timely recognize and take measures against the risk. This result indicates that physicians should be much more careful with regard to CIN development in patients with high H2FPEF score levels. Although the H2FPEF score is highly expected to have clinical value, large-scale long term studies are required to confirm its value.

 The present study had several limitations such as being a single center study, including only MI patients with acute ST elevation and not completely analyzing the potential nephrotoxic agents and lack of long-term results.

## Conclusion

 H2FPEF score is an independent predictor of contrast induced nephropathy development in patients with acute STEMI. H2FPEF score is practical and easy to calculate and implement in clinical practice and it may be used to estimate the CIN in STEMI patients. Use of the H2FPEF score may be helpful for being more careful and taking measures for prevention of CIN development.

## Author Contributions

**Conceptualization:** Ufuk Sadik Ceylan.

**Methodology:** Ufuk Sadik Ceylan.

**Validation:** Ufuk Sadik Ceylan, Ersin Yıldırım.

**Formal Analysis:** Ufuk Sadik Ceylan, Ersin Yıldırım.

**Iinvestigation:** Ufuk Sadik Ceylan, Ersin Yıldırım.

**Resources:** Ufuk Sadik Ceylan, Ersin Yıldırım.

**Data curation:** Ufuk Sadik Ceylan, Ersin Yıldırım.

**Writing-Original Draft Preparation:** Ufuk Sadik Ceylan, Ersin Yıldırım.

**Writing-Review and Editing:** Ufuk Sadik Ceylan, Ersin Yıldırım.

**Visualization:** Ufuk Sadik Ceylan, Ersin Yıldırım.

**Supervision:** Ufuk Sadik Ceylan, Ersin Yıldırım.

**Project Administration:** Ufuk Sadik Ceylan, Ersin Yıldırım.

## Funding

 The author (s) received no financial support for the research, authorship, and/or publication of this article.

## Ethical Approval

 This study was approved by Health Sciences University Haydarpasa Numune Training and Research Hospital Clinical Research Ethics Committee on 01 February 2021 with project number HNEAH-KAEK-2021/39-3303.

## Competing Interests

 The author (s) declared no potential conflicts of interest with respect to the research, authorship, and/or publication of this article.
